# The fixation paradox: when less is more in laparo-endoscopic inguinal hernia repair

**DOI:** 10.3389/jaws.2026.17808

**Published:** 2026-10-20

**Authors:** Mateusz Zamkowski, Maciej Śmietański

**Affiliations:** 1 2nd Department of Radiology, Medical University of Gdańsk, Gdańsk, Poland; 2 Śmietański Hernia Center, Lux Med Hospital in Gdańsk, Gdańsk, Poland

**Keywords:** inguinal hernia, M3 defect, mesh fixation, TAPP, TEP

## Introduction: we have been asking the wrong question

Laparo-endoscopic inguinal hernia repair has spent decades debating the wrong question. We ask whether the mesh should be fixed. That formulation assumes that the mesh is a neutral constant and that fixation is the decisive variable. Our experimental and clinical programme suggests the opposite. Preperitoneal meshes are not biomechanically interchangeable, and fixation is not an independent treatment decision. It is a downstream response to the mesh selected for a defined defect and to the quality of its deployment.

International guidelines already recommend non-fixation for most transabdominal preperitoneal (TAPP) and totally extraperitoneal (TEP) repairs, with large medial defects classified as M3 remaining the principal exception [1, 2]. Meta-analyses of randomised trials have not demonstrated an overall recurrence advantage from routine fixation in broadly selected laparo-endoscopic populations and suggest a possible pain penalty—one contributor to an outcome that is itself multifactorial—although M3-specific evidence remains limited [3, 4]. Registry studies further show that recurrence or reoperation risk is associated with the combination of defect class, mesh and fixation rather than with fixation alone [5, 6].

These data have usually been interpreted within the old binary framework: fixation or non-fixation. We believe that framework is now obsolete. The relevant question is not “Should this mesh be fixed?” but “Why is this the correct mesh for this defect?” Once a mesh with appropriate and demonstrated mechanical behaviour is correctly deployed in an adequate preperitoneal space, fixation has no instability to treat. If fixation is still chosen, the burden of explanation should lie with the surgeon who adds it.

## The hidden assumption: meshes are interchangeable

The fixation debate has been built on an unspoken assumption: that different meshes placed in the same anatomical plane are mechanically equivalent. They are not. A flat lightweight mesh, a rigid preformed implant and an individually shaped anatomical mesh may have similar surface area yet behave differently when exposed to medial pressure, folding forces, loss of pneumopreperitoneum and early patient movement.

Stability in TAPP and TEP is produced by the complete mesh–defect–technique construct: the mechanical demand of the defect; broad dissection and parietalisation; reduction of the sac and cord lipoma; mesh dimensions and effective overlap; geometry, bending behaviour, shape memory and conformity; correct orientation; and controlled peritoneal closure or desufflation. Fixation may be one component of a chosen strategy, but it is not the biological or mechanical endpoint. Patient-related and tissue factors act on every construct, fixed or unfixed. They modify risk; they do not make an unsuitable mesh suitable, and they are not an argument for fixation.

A fixed flat mesh can be an effective treatment. Its success, however, does not prove that the defect requires fixation. It establishes the effectiveness of that strategy, not the necessity of its fixation component. Conversely, an unfixed mesh is not safe merely because it is described as anatomical, three-dimensional or lightweight. The implant must be able to resist the forces generated by the defect for which it is used. Anchoring a poorly matched mesh does not make it the right mesh; it merely compensates for a construct that was not intrinsically stable.

## What our experimental programme revealed

Our pressure-chamber study exposed the variable that the fixation literature had largely overlooked [7]. Six spatial meshes and one flat macroporous mesh were tested without fixation in the same three-dimensional groin model containing an M3 defect. Heavyweight spatial meshes and a lightweight individually designed anatomical mesh maintained their position, whereas lightweight implants with insufficient rigidity—whether flat or preformed—migrated towards the medial defect. The defect, plane and absence of fixation were constant. Mesh behaviour changed. Under the tested conditions, the decisive variable was therefore the mesh–defect match, not fixation status.

The Individualized Laparoscopic Anatomical Mesh (ILAM) study extended this principle by asking whether stability could be engineered into the implant itself [8]. A computed-tomography-based, individually tailored mesh was designed to provide patient-specific overlap and multiple contact zones. The first implantation was uncomplicated and recurrence-free at 12 months. One case cannot establish effectiveness, but it demonstrates the concept: the stabilising function traditionally assigned to anchors can be incorporated into mesh geometry.

These experiments did more than show that non-fixation is possible. They explained why one unfixed mesh remains stable while another does not. This distinction changes the entire debate.

## MEFISTO: M3 does not mandate fixation

The MEFI protocol translated this hypothesis into a multicentre randomised trial [9]. MEFISTO enrolled 204 patients with M3 inguinal hernias in 12 Polish centres [10]. A fixation-free strategy using a rigid, anatomically shaped three-dimensional mesh was compared with a flat lightweight mesh fixed with tissue adhesive. At 12 months, recurrence occurred in 3.1% and 2.1% of repairs, respectively, meeting the criterion for non-inferiority; pain, operative time and surgical complications did not differ significantly.

MEFISTO was not a same-mesh fixation trial, and it should not be presented as one. That is not a weakness of its central message; it is the reason the trial matters. It compared two complete mesh–technique strategies and showed that M3 defects can be treated without fixation using the specific mesh–technique strategy tested. It did not isolate the independent contribution of mesh geometry or fixation.

The correct conclusion is not that every mesh may be left unfixed in every M3 defect. Our interpretation is more disruptive: M3 does not mandate fixation. M3 mandates an appropriate mesh. A large medial defect increases mechanical demand, but it does not dictate how that demand must be met. The statement “M3 requires fixation” confuses the properties of a defect with the limitations of a particular implant.

We argue that, after MEFISTO, invoking M3 alone is no longer an adequate justification for fixation. A surgeon who elects to add tackers, sutures or adhesive should be able to state what instability is being treated, why the selected mesh cannot provide the necessary stability, why a validated fixation-free alternative was not chosen and what evidence supports the added intervention. Routine fixation therefore requires a construct-specific justification, not defect classification alone.

## SWING-Mesh: the same mesh can be right and wrong

Our later work with new-generation anatomical meshes provides the most direct illustration of the mesh–defect principle. In pressure-chamber testing over an M3 defect, the lightweight preformed SWING-Mesh migrated at moderate pressure, whereas the medium-weight 3DMax MID Anatomical Mesh remained stable through the highest tested pressure [11]. The labels “anatomical”, “three-dimensional” and “lightweight” were therefore insufficient to predict performance.

M3 defects were deliberately excluded from the prospective multicentre clinical evaluation of fixation-free SWING-Mesh [12, 13]. The pressure-chamber findings support this indication boundary [11]. Among 294 non-M3 repairs—86.4% TAPP and 13.6% TEP—no recurrence was detected at 3 months [12]. At 12 months, 233 repairs were available for assessment and five recurrences were identified (2.15%); 93.1% of assessed cases were pain-free, although the 20.7% loss to follow-up limits certainty [13].

This is not a contradiction between laboratory and clinical evidence. We interpret it as support for the mesh–defect concept. The same mesh may be an appropriate fixation-free implant for one defect class and an inappropriate one for another. Asking whether SWING-Mesh “needs fixation” is therefore the wrong question. The question is whether SWING-Mesh is the right mesh for an M3 defect. Our experimental evidence indicates that it is not a validated fixation-free choice for that indication. For the studied non-M3 population, the clinical data support its use without fixation. The indication belongs to the mesh–defect pair, not to the mesh name alone.

## The mesh–defect–technique construct is the unit of evidence

We propose that the fundamental unit for both clinical decision-making and research should be the mesh–defect–technique construct (Figure 1). This includes a specifically identified mesh, its dimensions and relevant mechanical properties, the European Hernia Society defect class, the quality of preperitoneal dissection and parietalisation, the method of deployment and the manner in which the working space is released. Neither “fixation” nor “non-fixation” is meaningful when detached from this construct.

**FIGURE 1:**
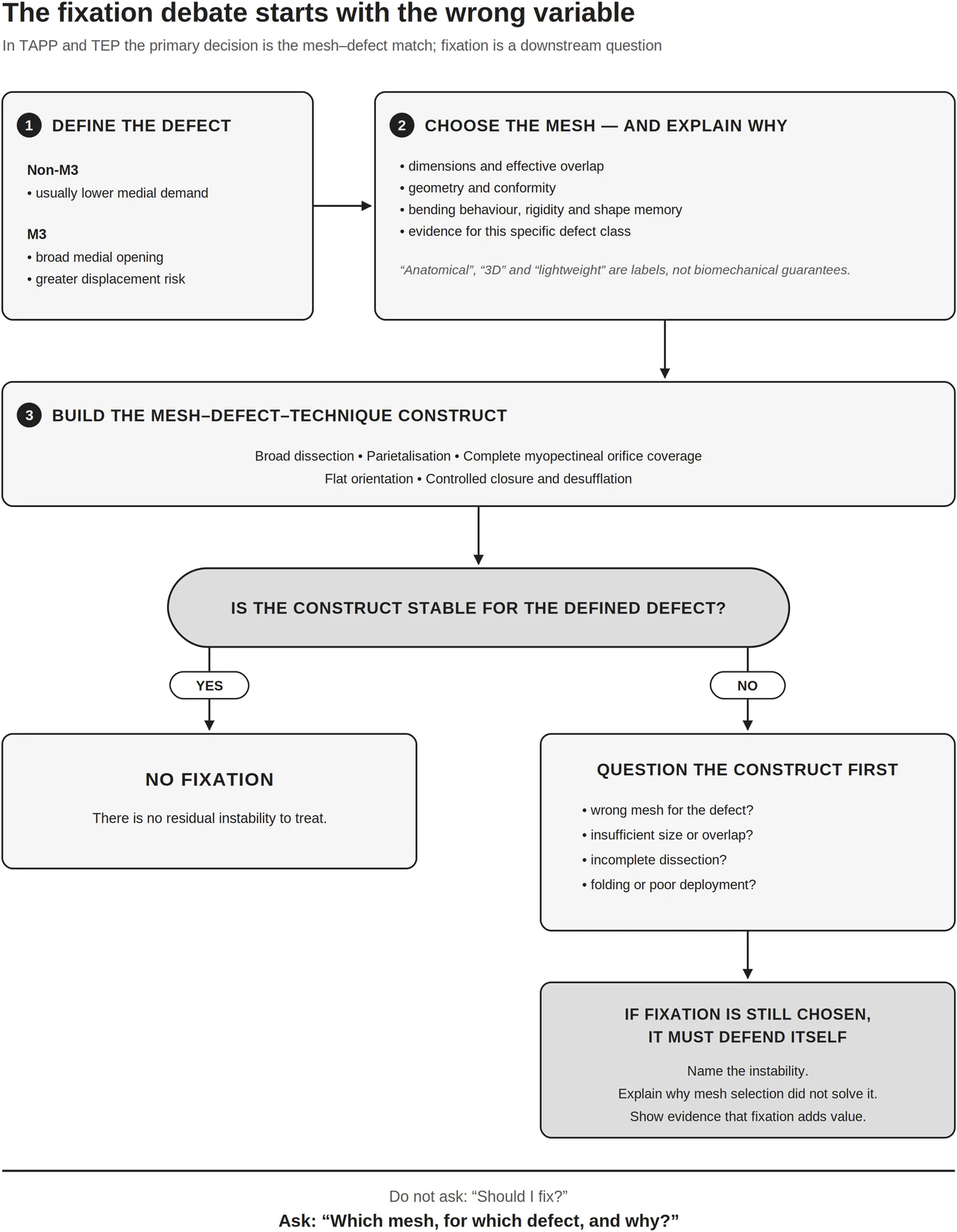
The fixation question is downstream of mesh selection. This conceptual framework presents the authors’ proposal; it is not a validated clinical algorithm. In transabdominal preperitoneal (TAPP) and totally extraperitoneal (TEP) inguinal hernia repair, the primary decision is the mesh–defect–technique construct. The defect defines mechanical demand; mesh dimensions, geometry, conformity, bending behaviour and defect-specific evidence determine whether the implant is appropriate; and correct dissection and deployment complete the construct. If the construct is stable, fixation has no instability to treat. If it is unstable, mesh choice, size, dissection and deployment should be questioned before an anchor is added. When fixation remains part of the strategy, it should be justified by a named mechanical deficit and evidence that it adds value. The proposed interpretation is that M3 demands an appropriate mesh–technique strategy rather than fixation solely because of defect class.

The practical sequence should therefore be reversed. First classify the defect and define its mechanical demand. Then select a mesh for which there is a rational and preferably experimental or clinical basis in that defect class. Create sufficient preperitoneal space and deploy the implant completely, flatly and in the correct orientation. If the construct is unstable, the first question should be whether the mesh, its size or the operation is wrong—not where to place a tacker.

Fixation may remain an intentional component of a validated strategy or an exceptional rescue measure. But it should no longer be treated as the default answer to uncertainty. We propose that future guidelines replace the M3 fixation exception with a requirement to demonstrate stability. This is a proposal for change, not a claim that current recommendations have already changed. Manufacturers claiming fixation-free use should demonstrate performance for the precise defect classes in which that claim is made. Trials should identify the mesh by trade name, dimensions, geometry and relevant mechanical characteristics, stratify by defect class, use standardised definitions of recurrence and chronic pain, and report TAPP and TEP separately. Same-mesh trials remain useful for isolating the effect of fixation, but strategy trials are necessary to compare the complete constructs surgeons actually use.

## Discussion: fixation must now defend itself

We are not arguing that fixation can never work. We are arguing that fixation is not the primary scientific question. Our translational programme—from pressure-chamber testing, through mesh design, to MEFISTO and defect-restricted clinical evaluation—has revealed the concealed variable: the mesh itself, matched to the defect.

The scope of this argument must remain explicit. The construct is a hypothesis and a research agenda, not a validated algorithm and not a guideline. Pressure-chamber stability does not establish long-term clinical effectiveness. MEFISTO compared strategies over 12 months, not the isolated effect of fixation; the SWING-Mesh cohort was uncontrolled and incompletely followed [10, 13]. These limitations constrain generalisation. They do not make different meshes interchangeable. Nor does the argument rest on pain: even a pain-neutral fixation would have to name the instability it treats.

The burden of proof should therefore reverse. Non-fixation with a correctly deployed, validated mesh–defect construct should not be required to defend itself against surgical habit. Fixation should defend itself. Every additional anchor should answer three questions: what specific instability is being treated; why was that instability not solved by appropriate mesh selection and deployment; and what evidence shows that fixation improves this particular construct?

The era of asking “Should I fix the mesh?” should end. The surgeon should ask: “Which mesh is appropriate for this defect, and why?” If that answer is mechanically and clinically sound, fixation usually becomes irrelevant. If fixation is still used, let it be justified.

*Open questions.* The construct generates questions the binary framework could not ask. Which mechanical properties—rigidity, bending stiffness, shape memory, effective overlap—predict the *in vivo* stability of an unfixed mesh in an M3 defect, and can a pressure-chamber threshold be validated against clinical recurrence? Does a fixation-free, mesh-matched strategy remain non-inferior beyond 5 years, and do late recurrences arise through mechanisms that fixation could have prevented? Does the same construct behave differently in TAPP and TEP? Can the fixation exception in guidelines be replaced by a demonstrated-stability criterion without loss of safety? Which patient and tissue factors, if any, change the answer?

Choose the mesh for the defect. Let the construct, not the anchor, provide the stability. If you still fix, name the instability you are treating.
